# Variation in Hsp70 Levels after Cold Shock: Signs of Evolutionary Responses to Thermal Selection among *Leptinotarsa decemlineata* Populations

**DOI:** 10.1371/journal.pone.0031446

**Published:** 2012-02-02

**Authors:** Anne Lyytinen, Johanna Mappes, Leena Lindström

**Affiliations:** Centre of Excellence in Evolutionary Research, Department of Biological and Environmental Science, University of Jyväskylä, Jyväskylä, Finland; Fred Hutchinson Cancer Research Center, United States of America

## Abstract

Individuals of widely spread species are expected to show local adaption in temperature tolerance as they encounter a range of thermal conditions. We tracked thermal adaptations of the Colorado potato beetle (*Leptinotarsa decemlineata*) that invaded Europe within the last 100 years. It has occupied various conditions although, like the majority of invasive species, it lost a measurable amount of neutral genetic variation due to bottleneck effect when it invaded Europe. We exposed diapausing beetles originated from three different latitudes (54°N, 59°N, 60°N) to cold shock (−5°C, 1.5 hrs) in order to test if beetles from the northern populations express differential levels of cold-induced and constitutive Hsp70 compared to the beetles from milder temperature regime. The level of cold-induced Hsp70 was lowest in the northernmost beetle populations while the level of constitutive Hsp70 did not differ with the population. Moreover, the southernmost beetles were more plastic in their response to cold shock than the northernmost beetles. These results suggest that physiological adaptation, like the synthesis of Hsp70, can evolve very quickly.

## Introduction

Why certain species occupy wide geographical areas whereas others are found in relatively small areas is a fundamental question in ecology. Especially harmful to insects are even brief exposures to sub-zero temperatures, i.e. cold shock, which can cause chill injuries [1]. Mortality is associated with protein denaturation [2], not due to internal ice formation as in freezing. Organisms have evolved several mechanisms to prevent these harmful changes. For example, heat shock proteins (Hsps, also referred to as stress proteins) are an example of adaptive phenotypic plasticity in inducible tolerance to environment. They can be induced by high or low temperatures [3] when they prevent aggregation of stress-denatured proteins and assist in refolding proteins to their native state [4], [5] thereby enhancing tolerance to various thermal stressors [6], [7].

Although the induction of Hsp synthesis by elevated temperatures has been intensively studied, only a few studies have focused on low temperatures [8]. Furthermore, as Karl et al. [9] pointed out, the role of Hsps in thermal adaptation has been investigated only in a few non-model species in terrestrial arthropods. The implication of the variation between conspecifics of different geographical origin for species distribution has not been widely assessed. Species also differ in whether Hsp70 is up-regulated during overwintering diapause but the reason for this interspecific variation is still unknown [10].

We studied the within species variation in the level of the Hsp70 in the diapausing Colorado potato beetles, *Leptinotarsa decemlineata* (Coleoptera: Chrysomelidae, Say), of different geographic origin. *L. decemlineata* is ideally suited for the investigation because it has gone through population bottleneck(s) while spreading from US to Europe, resulting in low neutral genetic variability [11]. Thus, it offers an opportunity to compare inter-populational differences and evolution of phenotypic plasticity in Hsp70 levels in species with low genetic variation. In such species phenotypic plasticity has been suggested to play a significant role in climatic adaptation [12]–[14]. In our experiment, diapausing beetles were exposed to ecologically relevant cold stress (−5°C) (see also [15]). Although *L. decemlineata* overwinters as an adult digging into the substrate, it still can be exposed to harmful environmental temperature. In the north of Europe, the depth of the frozen soil varies with year and place and, consequently, the optimum depth is unpredictable and exposure to cold is possible. We predicted that beetles originated from the north of Europe would express a lowered level of Hsp70. This is because Hsp production incurs significant costs albeit being beneficial [16], [17] and consequently organisms living in harsh environment might have evolved other, less costly mechanism to cope with cold [7], [18], [19].

## Materials and Methods

### Experiment on latitudinal variation

**Figure 1:**
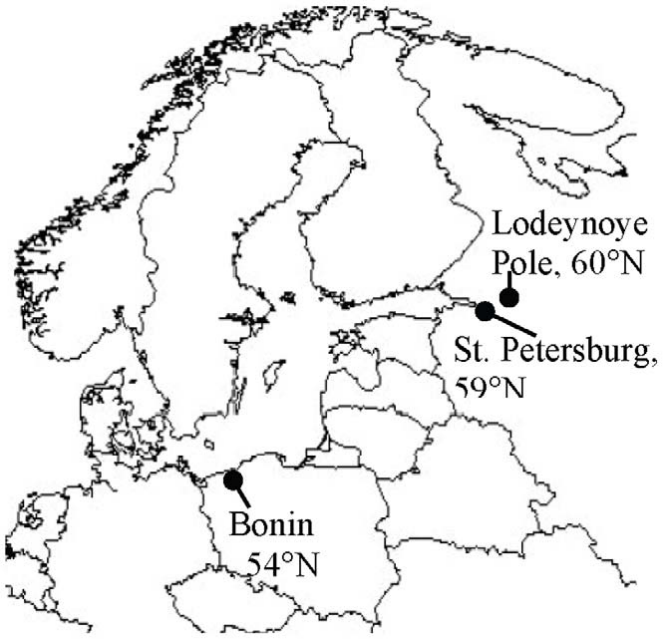
Collection sites of the beetles. The distance between south and north populations is about 1 300 km and the total north-to-south distance is about 700 km.

Colorado potato beetles originated from three different populations: Bonin (Poland, 54°09′N, 16°15′E), St. Petersburg (Russia, 59°54′N, 30°40′E), and Lodeynoye Pole (Russia, 60°44′N, 33°36′E) (Fig. 1). The distance between south and north populations is about 1 300 km and the total north-to-south distance is about 700 km. We could consider the populations to be separate ones because gene flow is low among European populations [11]. Collection sites vary in the mean monthly temperature and range of temperature in winter (Table 1). The beetles were imported and maintained in the laboratory under permission of the Finnish Safety Authority (Evira). The parent beetles that were descendents of field collected adult beetles overwintered in the laboratory after which they were allowed to lay eggs. Thus all the beetles used in the experiment were 2^nd^ laboratory generation, which might have changed Hsp70 levels (see [20]) but on the other hand, diminished the impact of maternal effects [21]. In order to test the hypothesis of the impact of laboratory rearing conditions, we also reared beetles from one population which were collected on two consecutive years (Lodeynoye Pole, Russia). Thus, one set of individuals was the second laboratory generation while the other set consisted of beetles hatched from the eggs that were collected in the same year as the experiment was performed. Larvae were reared in a greenhouse in cages on potted potato plants (variety Van Gogh) until emerging. Newly emerged adults were weighed, sexed, and moved to climate chambers under long-day conditions (16∶8, light∶dark) at 23°C. There beetles were allowed to feed on detached potato leaves (variety Van Gogh) for 2 weeks and then transferred individually to jars (4 cm diameter, 6 cm depth), filled with peat, and placed at the climate chamber in which diurnal mean temperature was 17°C in order to induce diapause. The temperature was 20°C during the 16 hours of the photophase, and 13°C during of the 4 hours of the darkness. The lights were dimmed gradually during 2 hours before and after the photophase, imitating the sunrise and sunset. During these hours, also the temperature slowly changed to the target temperature. When beetles had dug into the peat, they were transferred to the temperature of 5°C where they overwintered. If beetles did not dig into the soil within 10 day after transferring to 17°C, they were transferred first to 10°C for 5 days and then to 5°C.

**Table 1:** Minimum and maximum mean air temperatures and their average from nearest weather stations to the location of collection for *L. decemlineata*.

Collection site	Coordinates	Location of weather station	September–May (°C)*		
			Min	Max	Mean
Bonin,	54°09′N, 16°15′E	Koszalin	−3.9	17.3	5.1
Poland		(54.2°N, 16.2°E)			
St. Petersburg, Russia	59°54′N, 30°40′E	St. Petersburg (60.0°N, 30.3°E)	−10.7	15.6	1.2
Lodeynoye Pole, Russia	60°44′N, 33°36′E	Petrozavodsk (61.8°N, 34.3°E)	−15.0	13.3	−1.6

Bonin is situated in Koszalin, Poland. The collection site of Lodeynoye Pole (Russia) is located about 240 km northeast of St. Petersburg and Petrozavodsk. The data were obtained for Poland from www.weather.gov.hk and for Russia from www.worldweather.org.

*Note that these are the air temperatures. Temperatures at the overwintering depths of 50 cm below ground typically correlate with air temperatures but are a little higher.

### Cold shock treatment

The experiment was started when adult beetles had been in the overwintering diapause for 150 days. The sampling time corresponds to the midpoint of the diapause period in the northern part of the range. Half of beetles of each origin balanced with emergence weight was assigned to cold shock (Bonin: n = 8; St. Petersburg: n = 7; Lodeynoye Pole: n = 16) and control treatments (Bonin: n = 8; St. Petersburg: n = 8; Lodeynoye Pole: n = 14). In the cold shock treatment, beetles were exposed to −5°C for 1.5 hours, which is a sub-lethal temperature for *L. decemlineata* (none of the beetles died) and above their supercooling point [22]. After the cold shock they were allowed to recover at 5°C for 1.5 hours before being frozen in liquid nitrogen and stored at −80°C until Hsp70 analysis. In order to determine the constitutive level of Hsp70, control beetles were maintained at 5°C.

### Determining the total protein concentration

The beetles without elytra were weighed and homogenized in lysis buffer (1 mM EDTA, 0.5% Triton X-100, 10 µg/ml Leupeptin, 10 µg/ml Pepstatin, 100 µM phenylmethylsulfonyl fluoride (PMSF), 3 µg/ml Aprotinin in phosphate buffered saline, PBS). Homogenising was done on ice. After the homogenates were vortexed and left for 15 min on ice, they were centrifuged for 105 min at 16000 g at 4°C. The total protein concentration of the resulting supernatant was determined for 160 µl samples in triplicate (dilutions: 1∶500, 1∶1000, 1∶2000) by using a microassay procedure for microtiter plates according to the manufacturer's instructions (Bio-Rad Protein Assay). The reference protein samples were made of bovine serum albumin (BSA) diluted in PBS: 0, 5, 10, 20, 30, 40, 60, and 80 µg/ml. Absorbance was measured with microplate photometer (Multiskan Ascent, Thermo Labsystems). The samples were stored at −85°C until needed.

### Protein separation by SDS-PAGE

Proteins were separated by SDS-PAGE (Mini-PROTEAN Tetra Cell, Bio-Rad). The samples were diluted to a protein concentration of 2.5 mg/ml in PBS. Sample buffer was added to the samples in a ratio 1∶1 of the total volume to sample buffer and samples were denatured with sample buffer for 5 min at 95°C. An equal volume of each sample (2.5 mg/ml) and 50 ng/µl Hsp70 (recombinant human, NSP-555, StressGen Biotechnologies) were loaded on a gel. To control gel-to-gel variation, samples from each experimental group were divided among several gels and loaded on the gel in random order. High range of molecule weight standards (SDS-PAGE Molecular Weight Standards, Bio-Rad Laboratories) was also loaded on a gel. The gel was composed of 10% acrylamide separating gel (pH 8.8) topped by 5% stacking gel (pH 6.8). Electrophoresis was carried out at 80 V for 40 min and at 110 V for 3 h in electrophoresis buffer.

### Western blot analysis

**Figure 2:**
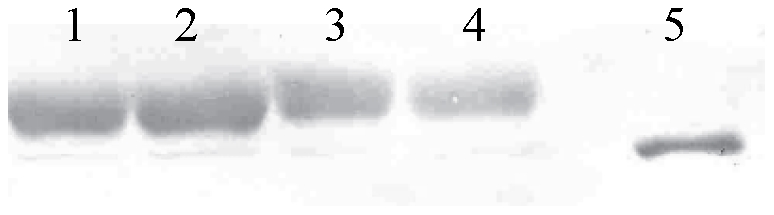
An example of Western blot analyses. Lines 1–2 show induced Hsp70 class protein, lines 3–4 constitutive Hsp70 and line 5 a 70 kD size.

Western blot assay was employed to detect the level of Hsp70 (Fig. 2). The samples were electroblotted onto nitrocellulose membrane (Whatman) according to the standard protocol. The blotting was carried out at constant current of 100 V in transfer buffer on ice for 2 hours. The blot was washed in 1×TBS for 5 min, stained with 0.2% Ponceau S for 5 min, and then rinsed briefly in de-ionized water. The blot was blocked with 5% non-fat dry milk TBS solution overnight at 4°C with constant shaking. Then blot was washed in TBS-Tween for 5 min and incubated with primary monoclonal antibody Hsp70 (1∶5000) in 5% non-fat dry milk TBS–Tween solution for 3 h with constant shaking. To locate Hsp70, mouse monoclonal anti-heat shock protein 70 (H-5147, Sigma, Missouri, USA) which reacts against Hsp70 and localizes both the constitutive (Hsp73) and inducible (Hsp72) forms of Hsp70. After the blot was washed three times in TBS-T for 5 min each, it was incubated with secondary antibody, Anti-Mouse IgG, for 2 h. The blot was washed three times in TBS-T for 5 min each. After washing, the blot was incubated at room temperature in APA buffer (pH 9.8) for 5 min. 5-bromo-4-cloro-3-indolyl phosphate (BCIP) and nitroblue tetrazolium (NBT) with APA buffer were used to visualize the bound antibodies. The gel was stained by Coomassie Brilliant Blue R-250 for 10 min and washed in 10% CH_3_COOH with constant shaking overnight.

### Determining the Hsp70 concentration

The intensity of blots was measured by ImageJ 1.40. Band size and intensities of samples were compared with bands of a serial dilution of pure Hsp70 (25, 37.5, and 50 ng/µl) loaded onto each gel. We used the serial dilution to create a standard curve and estimated the concentration of Hsp70 for each beetle by linear regression.

## Results

**Figure 3:**
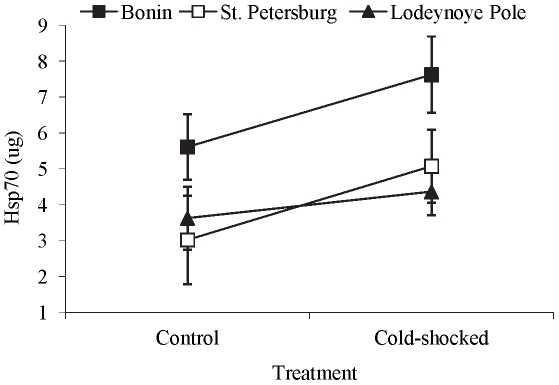
Hsp70 levels of the beetles. Reaction norms of Hsp70 levels (µg ± s.e.) in the control and cold shock treated beetles from three different populations: Poland (54°N), St. Petersburg (59°N), and Lodeynoye Pole (60°N).

As no significant differences in the Hsp70 level were found between the field and laboratory-derived populations from Lodeynoye Pole (2-way-ANOVA: F_1, 26_ = 0.305, p = 0.858), the two years were pooled for the further statistical analyses. A three-way ANOVA was employed to analyze the effects of the fixed factors treatment (control, cold shock), sex, and population (Bonin (54°N), St. Petersburg (59°N), Lodeynoye Pole (60°N)) on the Hsp70 level. Only interaction between the population and treatment was included in the final model. Other non-significant two- and three-way interactions (p≥0.275) were not included in the final model. There was no interaction between the three populations and cold treatment, indicating that all populations responded similarly to the cold shock treatment (3-way-ANOVA: F_2, 54_ = 0.355, p = 0.703; Fig. 3). This suggests that there is no change in the reaction norm among these three populations. The cold shock treatment increased Hsp70 (F_1, 54_ = 4.052, p = 0.049), without significant effects between males and females (F_1, 54_ = 3.539, p = 0.065). Hsp70 level varied more among populations (F_2, 54_ = 4.826, p = 0.012) with expression higher in beetles originating from the south (Poland, 54°N) than those from the north (LSD, St. Petersburg, (59°N): p = 0.015 or Lodeynoye Pole (60°N): p = 0.005). Hsp70 levels between the two northernmost populations, St Petersburg and Lodeynoye Pole, were the same (p = 0.996).

## Discussion

To occupy large geographical areas, organisms must evolve several mechanisms to avoid or tolerate stressful conditions. One mechanism to achieve stress tolerance is to synthesize Hsp70 as a response to a stressor. Diapausing *L. decemlineata* seemed to rely on this cold tolerance mechanism as it responded to thermal cold stress by up-regulating Hsp70 (see also [22]). The increase in Hsp70 level was more pronounced in the southern population that relatively seldom experiences such temperature than in the northern beetles. Thus our results lend support to the hypothesis that the production of Hsp70 as a response to temperature shock is less intense in organisms which are more frequently exposed to unfavorable temperatures in their habitat than those occupying benign conditions [7], [18], [19].

Beetles from different populations showed differential cold shock response. The populations have diverged from each other relatively recently; beetles arrived in Poland in 1950's [23] and Lodeynoye Pole (Russia) in 1990's [24]. Hence one might expect that insufficient time has elapsed since the populations diverged from each other to be able to detect inter-populational differences. Simply, due to the invasion history [11], one could have assumed that there would have not be enough genetic variability left to respond to selection to cold. In fact, the European populations have been shown to have low neutral genetic variability [11] and low additive genetic variability in physiological traits [25] although there is some genetic variability left in life-history traits [26], [27]. Differences in temperature exposure encountered during diapauses at the three sites are not large (Table 1) but the selection pressure seems to be strong enough to result in differences in cold response.

It is noteworthy that not all diapausing insects respond to stressor by upregulating Hsp70 [28], because Hsp production has been shown to incur significant costs [16], [17]. Inter-populational differences in Hsp70 upregulation shown in *L. decemlineata* might be a result of selection favoring other less costly mechanisms to cope with cold in the north as suggested in heat-shocked Drosophila flies [7], [18], [19]. One should note that there might be a mismatch between sampling time and the onset of Hsp70 synthesis which can differ with the severity of the shock [22] and the geographical origin of a population [29]. The severity of stress can also affect the Hsp70 level [22], [28]. We measured the Hsp70 level at only one time point and hence we might have missed the peak of the synthesis. We can rule out the potential effects of the laboratory rearing conditions on the threshold temperature for Hsp70 induction and the Hsp70 level [3], [20], [30], [31]. We collected eggs from the very same field in Russia (Lodeynoye Pole, 60°N) as the parental generation and reared them into adulthood in the laboratory. As no differences in the level of Hsp70 were observed between the laboratory-reared and field-collected populations, it is unlikely that the laboratory rearing conditions had major effects on Hsp70 level. Therefore the difference we observed in Hsp70 expression can best be explained by selection on the population due to climatic variation experienced by the beetles.
